# Clinical Remarks upon Surgical Cases in the Buffalo General Hospital—Operations for Strangulated Femoral Hernia and Fistula in Ano

**Published:** 1868-01

**Authors:** J. F. Miner


ART. II.—*Clinical Remarks upon Surgical Cases in the Buffalo General Hospital—Operations for Strangulated Femoral Hernia and Fistula in Ano.* BY J. F. MINER, M. D.

*Gentlemen:*

I am much gratified in being able to invite you to witness the operation for *Strangulated Femoral Hernia*—one of the most important forms of hernial protrusion, and one requiring operative interference, but very rarely in the practice of surgery. Yesterday I had opportunity to demonstrate to you the surgical anatomy of femoral hernia, and to show the different coverings of hernial protrusions, and the important relations of the internal epigastric and obturator arteries, calling your attention to the practical bearings which the relations of parts have in this operation. I am now able to show you this operation upon the patient, more forcibly impressing the practical lesson than could be done by demonstration elsewhere, however fully or frequently presented. This season you enjoy some of the rarest opportunities of combining the theoretical and practical in your education which were ever offered to medical students. Ovariotomy in the lecture-room and upon the operating table, femoral hernia in the lecture-room, both surgically and anatomically, and practically upon the operating table, are certainly forms of disease which circumstances rarely admit of presentation before classes in medical colleges, and on this account I have taken much pride and pleasure in being able to afford you such opportunity. You are but very infrequently to make either of these operations, but when they are required they are absolutely indispensable. The latter, when required, must be immediately and unhesitatingly made or the "golden opportunity" is lost, and death claims a victim, which it was your privilege and duty to save.

This patient, Mrs. D——, has what is called *strangulated femoral hernia.* The hernial tumor has been noticed for some time, but its true character unknown to the patient. Fourteen days since she was seized suddenly with great pain, referred to the same side as the hernia, but mostly to the bowels, and even as high as the umbilical region. For the last five days stercoraceous vomiting has been constant and very distressing. In this way the bowels have been relieved, which however unpleasant, was perhaps safer than accumulation of matter which should pass the other way. The appearance and history of the patient I am unable to give in detail, but when I saw her last evening the prostration, cold perspiration, vomiting, and as I learned, failure to obtain any dejections from the bowel, at once attracted my attention to an examination of the hernial regions with the expectation of finding either concealed, obscure, or fully formed hernia.

You will observe that the tumor is obvious and has the location, appearance, and general symptoms of femoral hernia. After making a little effort to reduce it by taxis, and finding it impossible to do so, and believing it unsafe to make very continuous or strong effort to do so on account of the unusual period since strangulation and the possible condition of the strangulated portion, I advised removal to the hospital with view of operation as soon as circumstances would permit, delaying a little in order that you might be present.

You will please observe that the incision is made in a direct line from above Poupart's ligament, extending over the prominent part of the protrusion. It is carried down through integument, superficial fascia, cribriform fascia, and the other less important coverings, to the peritoneum; the dissection being made carefully and without haste. The dark, congested, or inflamed appearance of this membrane contrasts strongly with the former tissues divided. The peritoneum, which constitutes what is called the hernial sac, is now opened, and an escape of serum shows that we have reached the cavity containing the strangulated intestine. Upon examination it is believed to still be capable of return to healthy condition, and that it should be returned to its natural place in the cavity of the abdomen. A grooved director is now passed through the constricted neck of the sac, and a small probe-pointed knife guided by it to the place of stricture, which is enlarged by simply turning the edge towards the parts to be divided, when, without much sawing motion the constriction is readily and sufficiently divided, and the intestine returns easily to its place. The sac has been protruded for a long time, and has formed connections with the adjoining parts, so that it cannot be returned. The stricture was formed by the sac itself, and not by the fibers of the fascia lata as they arch around and form the saphenous opening, which is the usual seat of stricture.

In this operation you see how the various coverings of hernia, so carefully demonstrated in surgical anatomy are scarcely distinguished at all, and you will naturally infer that this minuteness in anatomical detail is not observed in actual practice. This is wholly correct; yet you cannot but have been impressed with the importance of exact and full knowledge of the anatomical relations of parts, which is the essential and indispensable prerequisite to safe and successful operations for hernia. The successful return of the protrusion and the condition of the parts justifies the confident expectation of perfect recovery, though there are yet sources of danger.

In such case it will be sufficiently obvious from the single symptom of stercoraceous vomiting that there is some obstruction of the bowel; this symptom is not present in inflammation, and the cause is most frequently hernia; if no hernia can be detected, it should yet be considered if concealed hernia does not exist. If not hernia, *intussusception* of the intestine may next be suspected, and after this some very rare forms of obstruction are mentioned by authors, but all looking to mechanical obstruction to the natural evacuation of the bowels.

*Fistula in Ano.*—This case of fistula in ano presents nothing unusual in appearance. These fistulous tracts after a time are lined by a kind of false mucous membrane, which has no more tendency to unite with its fellow of the opposite side, than has other mucous surfaces. The causes of these tracts are obvious. Inflammation of the cellular tissue around the intestine, however caused, may eventuate in fistula — suppurative disease, opening first into the intestine, and afterwards increased and extended, opening externally forms a false passage. The operation consists simply in cutting from within outward all the superimposed tissue and allowing the parts to heal by granulation. The false mucous membrane is removed from the base of the incision, and the cure is usually complete. Time prevents further notice, but when I have opportunity to report results, I will complete what should be said upon fistula in ano.

